# Stress-Induced Visceral Pain: Toward Animal Models of Irritable-Bowel Syndrome and Associated Comorbidities

**DOI:** 10.3389/fpsyt.2015.00015

**Published:** 2015-02-16

**Authors:** Rachel D. Moloney, Siobhain M. O’Mahony, Timothy G. Dinan, John F. Cryan

**Affiliations:** ^1^Laboratory of Neurogastroenterology, Alimentary Pharmabiotic Centre, Biosciences Institute, University College Cork, Cork, Ireland; ^2^Department of Anatomy and Neuroscience, University College Cork, Cork, Ireland; ^3^Department of Psychiatry, University College Cork, Cork, Ireland

**Keywords:** visceral pain, stress, psychological, animal models, irritable-bowel syndrome, colorectal distension, microbiota–gut–brain axis

## Abstract

Visceral pain is a global term used to describe pain originating from the internal organs, which is distinct from somatic pain. It is a hallmark of functional gastrointestinal disorders such as irritable-bowel syndrome (IBS). Currently, the treatment strategies targeting visceral pain are unsatisfactory, with development of novel therapeutics hindered by a lack of detailed knowledge of the underlying mechanisms. Stress has long been implicated in the pathophysiology of visceral pain in both preclinical and clinical studies. Here, we discuss the complex etiology of visceral pain reviewing our current understanding in the context of the role of stress, gender, gut microbiota alterations, and immune functioning. Furthermore, we review the role of glutamate, GABA, and epigenetic mechanisms as possible therapeutic strategies for the treatment of visceral pain for which there is an unmet medical need. Moreover, we discuss the most widely described rodent models used to model visceral pain in the preclinical setting. The theory behind, and application of, animal models is key for both the understanding of underlying mechanisms and design of future therapeutic interventions. Taken together, it is apparent that stress-induced visceral pain and its psychiatric comorbidities, as typified by IBS, has a multifaceted etiology. Moreover, treatment strategies still lag far behind when compared to other pain modalities. The development of novel, effective, and specific therapeutics for the treatment of visceral pain has never been more pertinent.

## Introduction

Visceral pain is a severe form of pain that can be debilitating for the patient. Moreover, it affects a significant proportion of the population with up to 25% of people reporting visceral pain at any one time. Development of novel therapeutics is hindered by a lack of detailed knowledge of the underlying mechanisms, however progress is being made in this regard. The use of animal models has proved crucial in the advancement of our knowledge of what really is going on in visceral pain. This review aims to highlight the current state of play in the context of both preclinical and clinical research in the area of visceral pain. This review covers a broad range of research and as such in-depth details of studies is not included but is cited appropriately throughout. This review will summarize what is already known in the field and elude to future avenues yet to be explored in visceral pain research.

## Visceral Pain

Visceral pain is by definition, pain sensed as arising from the internal organs of the body (1). The pain may be described as sickening, deep, squeezing, and dull. Moreover, some organs are more sensitive to visceral pain than others (2). Diseases or disorders effecting certain organs such as the liver, lungs, or kidneys are commonly not associated with any overt symptoms of pain *per se* but mainly symptoms that are due to altered functioning of the organ itself. Conversely, other organs are far more sensitive to damage and can elicit excruciating pain. These organs include the stomach, bladder, and ureters (2, 3).

There are multiple etiologies for pain sensed in the internal organs, including: inflammation (acute and chronic), disruption of normal mechanical processes, neoplasms (benign or malignant), alterations in neurotransmission from the viscera, and ischemia (4–8).

Interestingly, visceral pain is intriguing in that pain is commonly felt in sites distant from the location of the organ itself. This referred pain, as it is known, is a key feature of visceral pain and is used by many clinicians in the diagnosis of certain diseases (1, 3). The pattern of pain sensation in referred pain can be similar across multiple organs and disease types, i.e., disorders of the gut, bladder, and other viscera are sensed as global abdominal pain, pelvic pain, or back pain, with specific localization very difficult to identify (3, 9, 10).

Visceral pain is the most common form of pain reported in the clinic and is the most common form of pain produced by disease (1). Although visceral pain is experienced by 25% of the population at any one time (11), in many cases it is insufficiently treated as it still remains to be considered as just a symptom of an underlying disease and not a disease in its own right. Over the last decades, the unsatisfactory treatment of visceral pain has led to an immense economic and personal cost, with patients experiencing a reduced quality of life and increased work absenteeism with escalating healthcare costs (12, 13). However, more recent literature suggests that novel pharmaco-therapeutic targets such as linaclotide (14) and μ-opioid receptor agonists and antagonists, selective κ-opioid receptor agonists, anti-inflammatory drugs, serotonergic agents, bile acid modulators, and intestinal bile acid transporters are performing well in clinical trials (15). To build momentum on these advances in clinical treatments, we must strive to enhance our understanding of the underlying mechanisms of visceral pain to aid future development of novel therapeutics. To fully appreciate the complexity of visceral pain processing, we must first understand the characteristics and neurobiology of this pain modality.

### Characteristics of Visceral Pain

As mentioned earlier, visceral pain perception and psychological processing is divergent to that of somatic pain (1). Importantly, there are clear distinctions which set visceral pain aside from all other pain modalities. These clinical features are crucial for the understanding of this complex physiological process. The characteristics of visceral pain were first outlined by Cervero and Laird (1) and have advanced our appreciation of this complex phenomenon. These characteristics are summarized in Table 1.

**Table 1:** **Characteristics of visceral pain [adapted from Cervero and Laird (1)]**.

Characteristics of visceral pain
1. Not all viscera have sensory innervation
2. It is not linked to visceral injury
3. It is referred to other locations
4. It is diffuse and poorly localized
5. It is accompanied by motor and autonomic reflexes

### Types of Visceral Pain

Visceral pain is the pain associated with a wide variety of disorders including gallstones, acute pancreatitis, acute appendicitis, diverticulitis, painful functional bowel syndromes such as irritable-bowel syndrome (IBS) and functional dyspepsia (FD), inflammatory bowel disease (IBD), gastroesophageal reflux disease (GERD), interstitial cystitis/bladder pain syndrome (IC/BPS), male chronic pelvic pain syndrome, and gynecological pain [endometriosis, vulvodynia, menstrual pain, polycystic ovary syndrome (PCOS)] (16–20). Moreover, and less commonly known is that visceral pain also encompasses chronic chest pain and colic (21, 22).

Appreciating this wide array of disorders has allowed us to understand the complex nature of the pathophysiology of visceral pain but have also induced considerable hurdles when aiming to fully understand the distinct subset of molecular mechanisms which underpin this phenomenon. For the remainder of this review, we will specifically discuss gastrointestinal (GI) visceral pain.

### Neuroanatomy of Visceral Pain

Knowing that the clinical characteristics of visceral pain are distinct from that of somatic pain, it implies that the neurobiology must also be distinct between these modalities. Indeed this appears to be the case with numerous groups reporting both anatomical and physiological differences (23–26).

**Figure 1:**
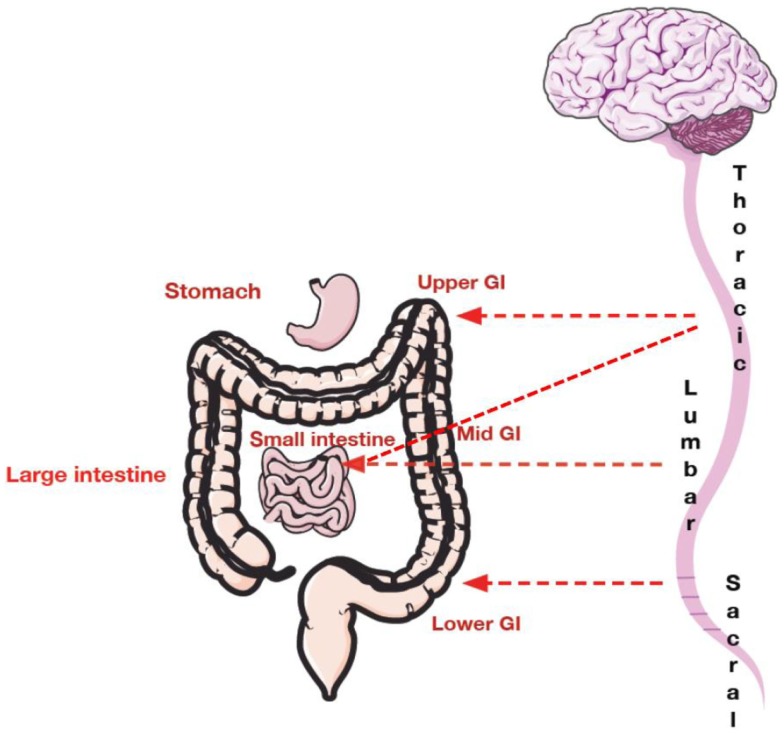
**Spinal innervation of the gastrointestinal tract**. The upper GI tract (including esophagus and stomach) is innervated with thoracic and lumbar afferents. The mid GI tract composed of the small intestine is innervated by both thoracic and lumbar afferents. The mid to lower GI tract including the large intestine is innervated by lower lumbar afferents and upper sacral afferents. The pelvic region is innervated by sacral afferents.

Pathways for visceral sensation are diffusely organized both peripherally and centrally. Communication of sensory information from the GI tract to the central nervous system (CNS) occurs via vagal, pelvic, and splanchnic nerve pathways (27–29). Vagal afferents innervate the GI tract from the esophagus to the transverse colon (8). Pelvic nerves innervate the remaining parts of the colon and rectum (8). A smaller group of afferents called the splanchnic afferents whose cell bodies arise from the thoracolumbar region of the spinal cord innervate the whole GI tract (27), Figure 1.

More specifically, primary afferent nerve fibers innervating the viscera, project into the CNS via three pathways: (1) the vagus nerve and its branches; (2) sympathetic pathways; and (3) the pelvic nerve (parasympathetic pathways) and its branches (26). Primary afferents signaling to the CNS reside primarily in the vagal nodose ganglion, which project to the nucleus tractus solitarius (NTS) located within the medulla of the brainstem (8), and in the T2–L2 and S1–5 dorsal root ganglia (30).

Visceral primary afferents have been demonstrated to enter the spinal cord and form synapses with dorsal horn neurons ipsilateral and contralateral to the site of entry. The result is extensive, diffuse CNS activation (31, 32). These axons form the postsynaptic dorsal column pathway. Interestingly, the dorsal column in itself has been shown to relay visceral nociceptive information and is now thought of as a visceral pain pathway in its own right. Numerous clinical studies have shown that lesioning the fibers of the dorsal columns significantly relieves pain and decreases analgesic requirements in patients suffering from cancer originating in the visceral organs (33, 34).

Visceral afferents terminate in laminae I, II_(outer)_, V–VII and X in the spinal cord. Laminae I and V form part of the spinothalamic tract, laminae VII and X constitute part of the dorsal column pathway (35, 36).

**Figure 2:**
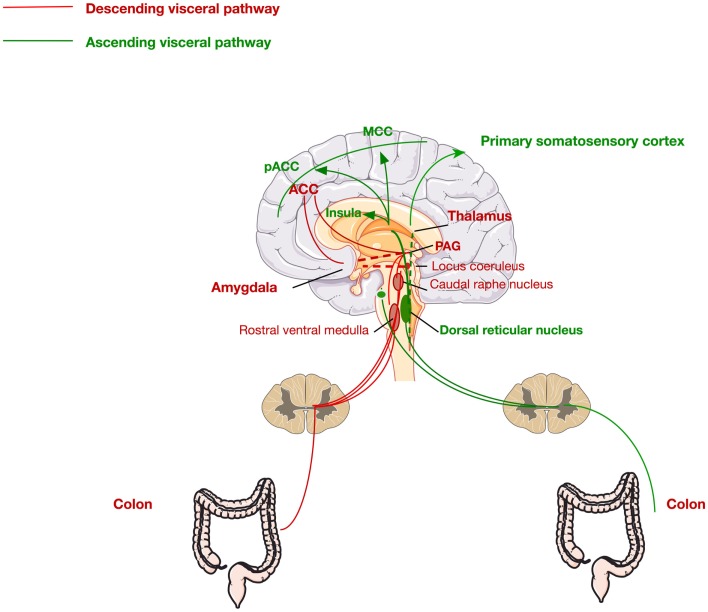
**Ascending and descending pathways mediating visceral pain sensation**. The ascending pathway for visceral pain perception from the periphery through the dorsal root ganglia via the dorsal reticular nucleus to the primary somatosensory cortex, insula, pregenual anterior cingulate cortex (pACC), and the midcingulate cortex (MCC). The descending pathway is mediated via signals from the ACC, thalamus, and amygdala to the periaqueductal gray (PAG), locus coeruleus, and raphe nucleus, returning via the rostral ventral medulla to the colon.

Second-order processing of visceral stimuli occurs at spinal segments and brainstem sites receiving primary afferent input. Figure 2 depicts the principal visceral afferent pathways projecting to the spinal cord, and then ascending to the thalamus and midbrain. These are the spinothalamic, spinoreticular, and spinomesencephalic tracts (37, 38). The spinothalamic tract transmits sensory information from the spinal cord to the reticular formation in the brainstem and terminates in the thalamus (medial and posterior) at which point the thalamocortical fibers project to the primary somatosensory cortex (38). This tract is responsible for sensory discrimination and localization of painful stimuli and thus the reflexive, affective, and motivational properties of noxious stimulation (38, 39).

The brain regions innervated by these pathways, more commonly known as the pain matrix, which are activated in response to colorectal stimulation include the prefrontal cortex (dorsolateral), the insula, the thalamus, the amygdala, and the anterior cingulate cortex (ACC) (38). The ACC is a critical component in the pain matrix and is functionally divided into two discrete regions, the perigenual ACC and the midcingulate cortex (MCC), with the former involved in affect and the latter in behavioral response modification (38, 40). Moreover, the amygdala, in particular, the central nucleus of the amygdala (CeA), is a critical region in the limbic system and the pain matrix. The CeA integrates many signals for the processing of painful stimuli. It receives input from the brainstem, as well as more complex information from the thalamus and the cerebral cortex. Moreover, it has also been shown to have direct synaptic interactions with locus coeruleus (LC) neurons (41), highlighting a clear role of stress pathways in the development of visceral pain. Furthermore, the amygdala is also known to receive afferents from the spinal cord through the spino-(trigemino-)parabrachio-amygdaloid pathway (42). It is also involved in the descending pain pathway and is involved in the emotional, affective, and cognitive functions of pain processing. Numerous preclinical studies have shown the important role of the amygdala in pain processing (43–52).

This multicomponent integration of nociceptive information explains the variability in the experience and reporting of visceral pain (37, 53) and thus the difficulty in development of effective pharmacological treatments. Interestingly, diffuse noxious inhibitory controls (DNIC) are a phenomenon more commonly referred to as “pain inhibiting pain” whereby a painful stimulus is applied to a part of the body, distant from the actual site of pain, thus inhibiting neurones within the dorsal horn of the spinal cord that are actively responding to chronic (unexplained) pain as seen in visceral hypersensitivity (54). DNIC is frequently used to quantify the central pain sensitization in chronic pain patients such as in the case of IBS (55–58). IBS patients consistently show a deficit in DNIC which correlates with symptom severity. These findings elude to the hypothesis that chronic pain patients are not only hyper-sensitive to pain but they also demonstrate reduced DNIC, possibly because of dysfunction of endogenous pain inhibition systems (55).

### Biochemical Mediators of Visceral Pain

Neurotransmitters, cytokines, and other mediators such as peptides and neuropeptides are thought to mediate visceral nociceptive signals from the periphery to the central pathways. Indeed, mediators released during peripheral inflammation and injury, are thought to influence spinal nociceptors resulting in increased nociceptive activity and central sensitization (27). For example, the inflammation and irritation associated with bladder infections is believed to cause the release of glutamate that sensitizes visceral primary afferents (59). Moreover, glutamatergic signaling in particular, metabotropic glutamate (mGlu) receptors and glutamate reuptake are fast becoming attractive areas of research in the context of visceral pain (60, 61). However, there are a whole host of other mediators and receptors that are involved in visceral pain processing including; neurotransmitter receptors [acetylcholine nicotinic receptors (62), cannabinoid receptors (63), opioid receptors (64, 65), GABA_A_, GABA_B_, and GABA_C_ receptors (66, 67), glutamate (ionotropic) receptors (68), glutamate (metabotropic) receptors (69, 70), glucocorticoid receptors (47, 71)], inflammatory receptors (bradykinin receptors, cholecystokinin receptors, cytokine receptors, leukotriene receptors, prostanoid receptors, tachykinin receptors, nitric oxide signaling, cyclooxygenase, lipoxygenase) (72, 73) and ion channel receptors [transient receptor potential vanilloid (TRPV) (74), purinergic (P2X) receptors (75), voltage-gated calcium channels (Ca_V_), voltage-gated potassium (K_V_) channels, voltage-gated sodium (Na_V_) channels] (76). Due to the vast array of mediators and pathways, a clear pathophysiology of visceral pain remains to be elucidated thus hindering drug development.

### Treatment of Visceral Pain

Although most visceral pain disorders are not life-threatening (non-cancer pain), they have a considerable negative impact on the quality of lives of patients with increased psychological distress, increased work absenteeism and both sleep and sexual dysfunction (77, 78). There are currently no pharmacological treatments on the market specifically for visceral pain. This leads to persistent bouts of discomfort and possible debilitation for the patient but also results in recurring visits to clinicians, associated with a significant economic burden on both the patient and healthcare services (12). Many patients are treated with multiple drug combinations to no avail. This void of effective analgesics in the context of visceral pain is frustrating for all parties involved. The drive to develop new analgesics has started right back to the basic molecular mechanisms of which very little is known (60, 79). Basic science and animal models have proved crucial in this effort for future developments of novel visceral analgesics.

In the clinical setting, treatment of visceral pain is extremely difficult. This is due to its complex nature, in that individuals can have many different triggering factors of their visceral pain and with no known cause, effective treatment strategies are difficult to identify. Treatment can differ from patient to patient and indeed treatment of the same patient over time may also change. As a result of this, a wide variety of pharmacological tools are used including a variety of analgesics [opioids (80), non-steroidal anti-inflammatory drugs (NSAIDS) (81), benzodiazepines (82)] and others (antibiotics, laxatives, serotonin modulators) (83, 84). Moreover, patients may also be treated with antispasmodics, particularly in the case of GI visceral pain and anti-depressants as well as others (84). Due to this heterogeneous pharmacological treatment profile, there are numerous and serious side effects including constipation, sedation, vomiting, tolerance, dependence, and addiction. However, none of the above are specific for the treatment of visceral pain *per se*, and mainly target some other features associated with chronic pain in general. However, in recent years, promising findings are emerging from both preclinical and clinical research which are nicely reviewed in Ref. (15, 83, 85, 86).

As with all chronic/severe pain disorders, opioids form the core of pain management for visceral pain conditions. However, as mentioned earlier, this class of analgesics are associated with the most serious side effects particularly over chronic use ranging from constipation (87) to analgesic tolerance (88–90) and nociceptive sensitization (91). More worryingly is the development of opioid-induced hyperalgesia after periods of prolonged opioid use (92–94).

Over the counter analgesics are routinely used by patients suffering with visceral pain. These include NSAIDS (aspirin, ibuprofen) and paracetamol. Again as with the case of most pharmacological agents used in the treatment of visceral pain, these agents do not specifically target visceral pain and only provide mild pain relief. Moreover, as these compounds are available over the counter they can be abused by consumers and carry a host of side effects not least GI bleeding, increased stomach acid, liver disease, liver failure, and even death in some cases (95).

Serotonergic compounds such as tegaserod (5-HT4 agonist) and alosetron (5-HT3 antagonist) (96–102) have been the main route of treatment for a range of visceral pain conditions, in particular IBS. However, tegaserod has since been removed from the market due to significant cardiovascular effects as outlined by the FDA. Alosetron was also withdrawn from the market in 2000 due to life-threatening adverse GI effects, however, in 2002, it was reintroduced but its availability and use were dramatically restricted.

Recent evidence from preclinical studies has pointed to a role of the transient receptor potential (TRP) channel family in the pathophysiology of visceral pain, which may lead to future development of novel therapeutics (103, 104). Moreover, both preclinical and clinical data have shown analgesic efficacy of pregabalin and gabapentin in acute and chronic visceral pain conditions, acting both at spinal and supra-spinal levels in particular at the level of the rostral ventromedial medulla (RVM) (60, 105–108). Furthermore, the efficacy of mGlu receptors and also glial glutamate transporters have revealed themselves to be very promising targets (59, 61, 70, 109–115).

As mentioned previously, there is an unmet need for effective pharmacological therapeutics in the context of visceral pain. However, there are still major challenges to be met, not least in the fact that we still lack a clear understanding of the etiopathogenesis of visceral pain disorders.

### Comorbidities of Visceral Pain

Psychiatric disturbances are the most frequent comorbidity of visceral pain (116–125). In particular, anxiety and depression are the most commonly reported comorbidities. This complex link between visceral sensation and psychological perceptions are mediated via the brain–gut axis.

Moreover, stress-related changes in bowel habit can attest to the fact that the brain can influence gut function and sensation (126). Several clinical studies have suggested that psychosocial comorbidity is a major contributor to the severity and impact on quality of life of visceral pain disorders such as IBS and somatic pain disorders such as fibromyalgia (127–131). These findings are reinforced by a considerable volume of experimental research that links stress, anxiety, and depression to altered GI sensory and motor function as well as altered pain processing (8, 132–138). Indeed, successful management of patients with visceral pain disorders requires careful attention to these psychosocial factors, often in consultation with mental health professionals.

### Pathophysiology of Visceral Pain

The etiology of visceral pain is most likely multi-factorial involving biological, psychological, and social factors leading many to describe visceral pain as a biopsychosocial disorder. Due to the array of comorbidities associated with visceral pain, it is clear that both the brain and viscera play significant roles through the brain–gut axis (139–142). Moreover, the emerging role of the gut microbiota on brain signaling has now led to the concept of the microbiota–brain–gut axis (143–145). Numerous other pathways and systems feed into this complex network of communication, including the stress axis and immune system. The role of the amygdala in IBS has been extensively reviewed in the context of brain–gut axis communication (146) and numerous clinical and preclinical trials have also highlighted an important contribution of the amygdala to visceral pain processing and IBS (52, 147, 148). Here, we discuss the pathophysiology of visceral pain in terms of signaling along the microbiota–brain–gut axis, the hypothalamic–pituitary–adrenal (HPA) axis, and the immune system. Many other factors such as gender, genetics, and epigenetics are implicated in these pathways which not only exacerbate visceral hyperalgesia but may also be predisposing factors for the development of visceral hypersensitivity in later life. Furthermore, we also discuss possible future targets for visceral analgesia including the modulation of mGlu receptors in addition to glial glutamate transporters and histone deacetylation.

## Stress

Stress was first described by Hans Selye almost 80 years ago, and is defined as an acute threat to the homeostasis of an organism (149). Stressors can be in the form of physical threats such as an adverse event, or psychological stressor, such as anticipation of a threat. Exposure to these stressors will elicit a sequence of physiological, emotional, and behavioral reactions that allow one to cope adequately with the situation (150–152). Behavioral effects of the stress response include increased awareness, improved cognition, and altered pain sensitivity. Physiological adaptations include increased cardiovascular function, enhanced respiratory rate, and altered metabolism, along with inhibition of feeding, digestion, growth, reproduction, and immunity (153, 154).

The complexity of the sequence of responses to stress involves a range of systems including endocrine, nervous, and immune systems. The efficiency of this response ensures that the correct behavioral and physiological changes occur so that the individual responds appropriately to the stressor presented and improves the individual’s chance of survival (155, 156). Understandably, due to the considerable complexity of the stress response, a host of regulatory mechanisms are at play to ensure the stress response is tightly regulated and does not become pathogenic to the host. These regulatory mechanisms are present at all levels of the stress response but particularly so at the neuronal and endocrine level which function to tightly regulate this adaptive process (157). However, the body can also elicit maladaptive changes in brain structure and function in response to chronic and uncontrollable stressors, thus, leaving long-lasting signatures on global wellbeing (158–160).

Dysregulation of the stress response has been associated with a plethora of disorders and diseases including chronic and visceral pain, autoimmune diseases, hypertension, affective disorders, and major depression (8, 161). Deciphering the pathogenesis of such disorders and their aberrant regulatory mechanisms will aid future therapeutic strategies for treatment and prophylaxis of stress-related disorders including stress-induced visceral hypersensitivity (154).

### The Hypothalamic–Pituitary–Adrenal Axis

The HPA axis is the main stress axis in mammals and its anatomical structures are located both in the CNS and in the periphery. The major components of the stress axis are localized in the paraventricular nucleus (PVN) of the hypothalamus, the pituitary gland (anterior lobe), and the adrenal gland (154). In response to stress, corticotrophin-releasing hormone (CRH) is released and travels to the anterior pituitary gland where binding of CRH to CRH receptors (CRHR1 and CRHR2) leads to the release of adrenocorticotropic hormone (ACTH) into systemic circulation. ACTH targets the adrenal cortex to produce and secrete glucocorticoids (154). Glucocorticoids are the main effector molecules of the stress response and regulate the physiological adaptations through binding to their intracellular receptors (162, 163). Dysregulation of the HPA axis via inadequate or excessive activation, is thought to contribute to the development of a wide array of pathologies (154, 162, 164). Indeed CRH and its receptors have been extensively investigated in the context of stress and visceral pain (165–177). Taken together, it is apparent that stringent regulation of the HPA axis is essential to a normal adaptive and efficient stress response.

## Stress and Irritable-Bowel Syndrome

The association between stress and psychiatric disorders, is well known, however, what exact molecular changes occur to underpin this increased vulnerability to disease is on-going (145, 178). Psychiatric disorders in addition to stressful life events are predisposing factors for the development of functional gastrointestinal disorders (FGIDs) such as IBS. IBS is one of the most common FGIDs with an estimated prevalence of 10–20% (179). Symptoms include abdominal pain, altered stool consistency and frequency, bloating, and distension. The pathophysiology of IBS has implicated stress as one the most significant risk factors for the development of the disorder (180–183). Stress at different stages throughout life, and especially early in life, can have deleterious effects on both psychological wellbeing and GI function of the host (Figure 5). Figure 5 depicts the roles of stress (vulnerability, trigger, perpetuating) in IBS pathophysiology at critical points throughout life.

Over the last decade, there has been an abundance of reports implicating stress in the onset or exacerbation of symptoms of IBS (182, 184–186). Moreover, in a preclinical setting, animal models of IBS are predominantly stress-based models (187) aimed at elucidating biomarkers of this complex biopsychosocial disorder. If we look at stress throughout our life, some critical developmental windows such as early life and adolescence are associated with the development of a wide variety of disorders, not least visceral pain disorders such as IBS. Stress, particularly during early life can manifest in many different forms including physical trauma, loss of a parent, abuse (physical/sexual), all of which have been associated with an increased risk of developing FGIDs later in life (188, 189). Furthermore, acute stressors such as sexual abuse, rape, traumatic event (near fatal event), and warfare are also risk factors for the development of IBS (122). Individuals responses to stress vary, a phenomenon thought to be based both on genetic and epigenetic mechanisms (180). The area of stress susceptibility and stress resilience is of interest across all areas of psychiatry (190, 191) and also in the context of comorbidities such as chronic pain disorders (192). Chronic stress may alter individual’s responses and play a strong role in symptom exacerbation. For example, psychosocial stressors in the form of sustained, threatening life events have been associated with onset and symptom exacerbation in IBS (181, 182, 193–195). Moreover, chronic stress has also been shown to induce adaptive changes in neuronal circuitry at the level of the CRH receptor 1 expression in the PVN of the rat hypothalamus, with reduced CRHR1 expression in comparison to acutely stressed rats after repeated homotypic stress exposures (196).

Differing coping strategies employed by patients, have also been shown to have a profound effect on symptom severity due in part to adaptation to stress (197–200). These studies highlight the high degree of catastrophizing or maladaptive coping within IBS groups. Indeed, positive coping strategies were shown to have positive effects on perceived stress levels and symptoms, however biomarkers such as cortisol remained unchanged. Furthermore, other functional somatic syndromes such as fibromyalgia are also known to exacerbate IBS symptoms (201, 202).

Psychological stressors are not the only risk factor for the development of IBS, but also physical stressors such as infection. Recent evidence from a systematic review and meta-analysis demonstrated that there was a sixfold increase in the risk of developing IBS after GI infection. Moreover, this enhanced risk remained elevated for at least 2–3 years post-infection (203, 204). The vicious cycle that stress plays in terms of vulnerability, trigger and perpetuation is most notable in patients who have developed IBS and associate particular situations, surroundings, or scenarios as provoking factors. This type of fear conditioning plays a key role in triggering stress responses to situations and contexts that by themselves are not threatening or stressful (205). In the context of IBS patients, this forward drive of conditioned fear-responses to both physical stimuli (infection) or contextual stimuli (location of stressful event) may be a significant factor in symptom chronicity (182). Using this knowledge and applying it to animal models will be discussed in later sections (206).

## Microbiota and Visceral Pain

### The microbiota–brain–gut axis

**Figure 3:**
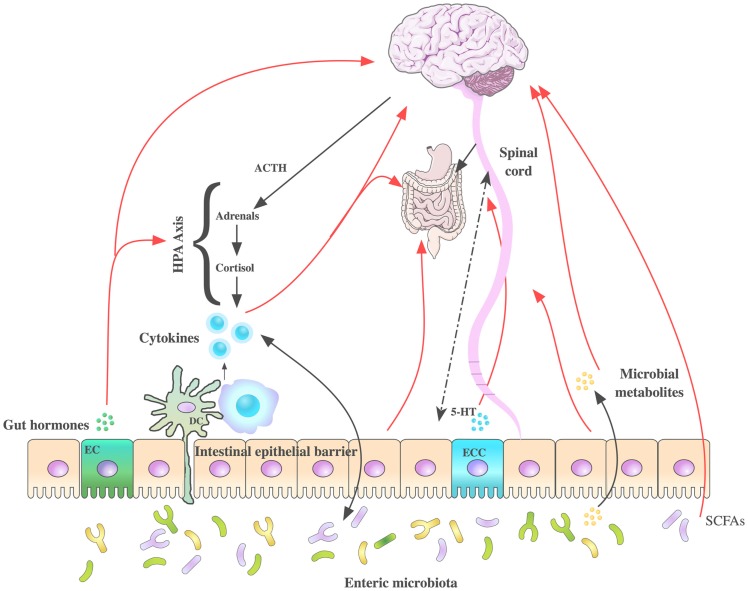
**Routes of communication along the microbiota– brain–gut axis**. Several pathways have been proposed to understand the communication between the intestinal microbiota and brain function, some of which have been summarized in this figure. These include neuroendocrine (hypothalamic–pituitary–adrenal axis), immune system (neuromodulating cytokines), enteric nervous system, autonomic nervous systems (vagus nerve), and spinal afferents. 5-hydroxytryptamine (5-HT) is produced by enterochromaffin cells in the GI tract. Gut microbes produce tryptophan-related metabolites, gut hormones, short chain fatty acids (SCFAs), and neurometabolites GABA, noradrenaline, and dopamine potentially modulating CNS function. Stress can influence the microbial composition of the intestine through the release of stress hormones (corticosterone/cortisol) or sympathetic neurotransmitters that in turn can influence gut physiology and alter the microflora balance. DC, dendritic cell; EC, enteroendocrine cells; ECC, enterochromaffin cells.

The bidirectional signaling network that exists between the GI tract and the brain is vital for maintaining homeostasis and is regulated at the neural [both central and enteric nervous systems (ENS)], hormonal, and immunological levels. Perturbation of these systems results in alterations in the stress response and overall behavior (143, 207). The high rate of psychiatric comorbidities with GI disorders and vice versa (208–210) are further evidence of the importance of this network of communication. The brain–gut axis is by no means a new discovery however its role in many disorders outside of gastroenterology has become an area of intense research. Moreover, advances in biomedical research have allowed us to elucidate the role of the gut microbiota community in signaling along this axis, which is now more commonly referred to as the microbiota–brain–gut axis (Figure 3). The impact of the microbiota–brain–gut axis has become a rapidly advancing research topic encompassing broad domains of biomedical research including neuroscience, psychiatry, gastroenterology, and microbiology.

The classical construct of the brain–gut axis, describes the way in which complex bidirectional signals can transmit between the CNS and the GI tract. This axis is critical for preserving gut homeostasis, dysregulation of which has been implicated in an array of disorders and disease states including gut inflammation, chronic abdominal pain syndromes, and eating disorders (143–145, 210–217). The brain–gut axis is responsible for some of our most basic functions such as facilitating the central regulation of digestive function. Indeed, the role of this axis is a well-developed concept in the area of food intake and satiety (218, 219) and more recently obesity (220, 221).

Moreover, the role of the microbiota–brain–gut axis in the stress response via modulation of the HPA axis has been investigated by numerous independent research groups (222–228). Furthermore, many forms of psychological stress have been shown to alter the gut microbiota fingerprint with many prebiotics and probiotics showing beneficial effects against the negative impact of stress. Thus, it is now acknowledged that the gut microbiota themselves are critical mediators of information along this bidirectional communication network (145, 207, 211, 213, 229, 230).

Indeed, in IBS patient cohorts, numerous independent research groups have shown distinct gut microbiota populations when compared to healthy controls (231–236). This was recently reviewed by Mayer and colleagues (237). Moreover, probiotic interventions appear to be beneficial to IBS patients (238).

Manipulation of the gut microbiota through the use of probiotic and prebiotics treatments have shown that by augmenting so-called “good bacteria” such as *Bifidobacteria* and *Lactobacillus*, in the gut, visceral hypersensitivity can be reversed in preclinical models. A mixture of eight probiotic bacteria strains (VSL#3) was shown to have protective effects against development of visceral hypersensitivity in the neonatal maternal separation model. Moreover, TPH1, tryptophan hydroxylase 1, the gene for the enzyme responsible for synthesizing serotonin, a key neurotransmitter involved in IBS treatment, was markedly up-regulated by neonatal maternal separation and this effect was reversed by VSL#3 intervention (239). Moreover, the same cocktail of probiotics was shown to prevent visceral hypersensitivity induced by inflammation via intra-colonic instillation of 4% acetic acid when given prophylactically (240). *Bifidobacterium* species particularly, *Bifidobacterium infantis* 35624 has been shown to be particularly effective at ameliorating visceral hyperalgesia in both stress-induced visceral hypersensitivity and colitis (241–243). Moreover, *Lactobacillus* species have also displayed efficacy in visceral pain models (244–247).

Furthermore, antibiotic-induced visceral hypersensitivity again underpins a role of the gut microbiota in the pathophysiology of visceral pain (244, 248). Interestingly, rifaximin, a semisynthetic, non-absorbable antibiotic that demonstrates no clinically relevant bacterial resistance has also shown positive effects in the treatment of IBS (249–257). These findings may seem contradictory, however, rifaximin is particularly efficacious in cases of small bowel bacterial overgrowth found in IBS patients. These findings add to the growing literature that microbiota dysfunction may be a key player in the pathophysiology of IBS and may lead to future novel therapeutic interventions.

## Immune System and Visceral Pain

The immune system and thus inflammation have long been associated with psychiatric disorders, in particular, depression (258–260) and chronic pain disorders (261). Depression is a common comorbidity of visceral pain, as discussed earlier, so it is not surprising that a common mechanism such as neuroinflammation may be at play. The immune system is a critical component of the microbiota–brain–gut axis and plays vital roles in maintaining homeostasis in the nervous systems and GI tract (262). Moreover, direct communication occurs between the immune system and the HPA axis, autonomic nervous system (ANS) and ENS (263–267). These integrated pathways are all known to be involved in the pathophysiology of visceral pain, and thus it is not unforeseen that the immune system plays a key role in the development of visceral hypersensitivity.

### Microglia and Visceral Pain

Microglia represent the first line of defense for the CNS, acting as a sensor for pathological events (268). The process of central sensitization and the subsequent changes in synaptic plasticity has long been thought to play a major role in nociceptive processes both in the context of chronic pain as well as acute, in both somatic and visceral modalities (269, 270). In the last decade, the role of microglia, both spinal and supra-spinal, has become an area of interest in the context of nociception (271–273). In animal models of both inflammatory and neuropathic pain, activation of microglia is a key step in the onset and maintenance of hypersensitivity and allodynia (274–279).

The role of spinal microglia in visceral pain has only recently been addressed and reviewed nicely by Lu (269). Saab and colleagues were first to report increased activated microglia in a rat model of chronic visceral hyperalgesia, namely the neonatal colon irritation model (280). Moreover, minocycline, a second-generation tetracycline compound known to interrupt microglia activation and its associated pro-inflammatory response, reversed the visceral hypersensitivity in adulthood (269, 280).

Furthermore, enhanced microglia activation was reported in the hippocampus, in a model of trinitrobenzene sulfonic acid (TNBS)-induced colitis, concomitant with increased tumor necrosis factor-α (281). However, this study did not assess nociceptive behavior, but one could predict visceral hyperalgesia as observed by others in the TNBS-model (282). More recently, it was shown that the activation of spinal microglia plays a critical role in the initiation and maintenance of visceral hypersensitivity in the TNBS-induced chronic pancreatitis (CP) rat model (283). Intrathecal injection of minocycline attenuated visceral hypersensitivity and phospho-p38 levels in CP rats.

In other non-inflammatory models of visceral hyperalgesia, it has also been shown that chronic psychological stress leads to microglia activation in the lumbar spinal cord (284). Moreover, this stress-induced increase in activated microglia could be blocked by SB203580, a p38 inhibitor or minocycline. Other compounds known to alter the microglia phenotype such as FKN, a microglia activator, was shown to induce visceral hypersensitivity in naïve rats when administered spinally, an effect which was blocked by minocycline (269), thus adding further evidence to support the role of spinal microglia in mediating stress-induced visceral hypersensitivity (269). Taken together, these studies suggest that microglia may play an important role in the pathogenesis of visceral pain.

However, it is also pertinent to note that exact role of inflammation and indeed micro-inflammation still remains controversial in the context of IBS. Indeed, no effect of anti-inflammatory drugs has been shown in IBS such as prednisolone (285).

### Toll-Like Receptors as Novel Therapeutic Targets for Visceral Analgesia

**Figure 4:**
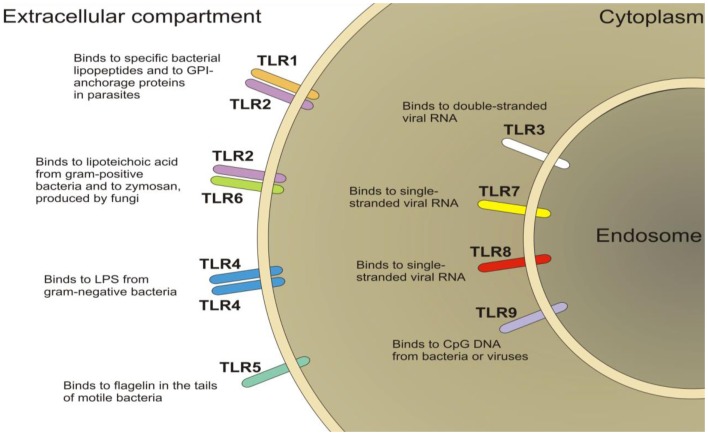
**Schematic of the localization of toll-like receptors (TLRs)**. TLRs are located on the plasma membrane (TLR1, TLR2, TLR4, TLR5, TLR6, TLR10) with the exception of TLR3, TLR7, TLR8, and TLR9, which are localized in the endosomal compartment.

Toll-like receptors (TLRs) are a family of pattern-recognition receptors of the innate immune system (Figure 4). There are 10 human TLRs and 12 mouse TLRs (286). TLR signaling consists of at least two distinct pathways: (1) the MyD88-dependent pathway, which leads to a pro-inflammatory phenotype, and (2) the MyD88-independent pathway, which leads to the production of interferon-β and the maturation of dendritic cells. The MyD88-dependent pathway is common to all TLRs, except TLR3 (287). TLRs are important players in the maintenance of mucosal and commensal homeostasis within the gut via innate host defense mechanisms. Intestinal dysbiosis and inflammation underlie several disease states affecting the GI tract such as IBS and IBD (288, 289). Indeed, reports have shown the involvement of peripheral TLR4 in patients suffering from IBS (290, 291) and in animal models of visceral pain (289, 292, 293). Moreover, the presence of TLR4 in the ENS and in the dorsal root ganglia indicate a role for TLR4 in the transmission of sensory information from the GI tract (294, 295). Furthermore, TLR4 is also expressed within the CNS, predominately in microglia (296), which have been discussed above and their role in visceral pain. Moreover, it is now emerging as a possible therapeutic target for neuropathic pain (297). Taken together, these findings suggest TLR4 as a promising novel target for the treatment of visceral pain.

#### Interaction of TLRs and Opioid Receptors

In recent years, it has been shown using *in vivo, in vitro*, and *in silico* techniques that members of each structural class of opioids (μ, κ, δ) activate TLR4 (298). Moreover, opioid antagonists such as naloxone and naltrexone non-stereoselectively block TLR4 signaling (299, 300). Modulation of TLR4 expression/function by acute blockade of TLR4, genetic knockout of TLR4, or blockade of TLR4 downstream signaling each lead to a potentiation of the magnitude and duration of opioid analgesia (301). These effects are thought to be mediated at both spinal and supra-spinal sites (299). Given the breadth of opioids now documented to interact with TLR4, many off-target opioid effects previously attributed to unilateral opioid action at classical neuronal opioid receptors might in fact result, at least in part, from the duality of opioid actions at TLR4 (301).

## Gender and Visceral Pain

Sex differences in pain sensitivity has been a topic of debate for many years and was recently reviewed by Mogil (302). More recently, gender differences in visceral pain and in particular IBS have been reviewed in Ref. (303) and indeed the contribution of sex hormones (304). Moreover, we can now appreciate that gender differences are also apparent in analgesic response (305). Many forms of visceral pain due to their nature are especially prevalent in women, i.e., pain associated with reproductive function (menstruation pains, pain of child birth, or postmenopausal pelvic pain). IBS is a disorder predominated by females (female to male ratio ~ 2:1) which is in parallel with women also being more susceptible to stress-related disorders (306, 307). Indeed, many studies have reported sex differences in the stress response itself and stress-induced pain modulation (8, 308). However, in the case of animal studies, the picture is not as clear. This is due in part to a bias for using male rodents in most preclinical studies addressing the role of stress modulation on visceral hypersensitivity (134, 135, 289, 309–311).

Sex/gender can influence the brain–gut axis, at an array of sites which logically will effect subsequent clinical outcomes including response to behavioral and drug therapies in patients suffering with visceral hypersensitivity (312). A plethora of factors including mood, stress, gender role, hormones, as well as inflammatory mediators modulate the brain–gut axis (313). As a result, the explanation for gender-related differences in visceral pain is likely multi-factorial involving environmental, psychological, and biological (sex) influences (312, 314, 315).

Studying gender differences in any context comes with significant difficulties, not least in the context of visceral pain disorders. Due to the greater number of women who are diagnosed with these disorders, there is often insufficient power to detect differences in the etiologic factors and treatment response by gender or sex. For example, several studies examining the effectiveness of cognitive behavioral therapy in reducing symptom distress have included only women (316, 317) or were disproportionately composed of women (318). In particular, the smaller number of men in drug trials leaves many studies underpowered to examine gender differences (313).

The variability in reproductive hormones such as estrogen and progesterone, across the menstrual cycle as well as significantly reduced ovarian function during and after menopause may explain changes in GI motility and visceral sensitivity (319). This has been reported in several studies documenting the impact of menstrual cycle on GI symptom reporting including visceral pain (320–323). The most consistent finding is that, many women with and without FGIDs experience an increase in GI symptoms including visceral pain during the late luteal and menses phases of the cycle relative to other cycle phases (313, 323–325). To add to the complexity, is the addition of pharmacological agents known to impact on the menstrual cycle and associated functions, such as oral contraceptive use and hormone replacement therapy. Moreover, the presence of other reproductive organ problems unique to women such as dysmenorrhea can also confound such studies.

It has been well established that adult females have higher basal and stress levels of ACTH and corticosterone than do males (326–330). Moreover, there is convergent lines of evidence reporting that gonadal hormones, specifically the estrogens, are important regulators of the HPA axis. Indeed, estrogen receptors α and β themselves have been shown to increase CRH expression (8, 331, 332). Females have higher CRH, ACTH, and corticosterone levels during proestrus (333), the phase of the cycle in which estradiol levels are highest, than during other phases of the estrous cycle (334–336). Fluctuations in gonadal hormones specifically, estradiol, during the menstrual cycle can also lead to changes in the neurotransmitter systems, in particular, the serotonergic and glutamatergic systems. Interactions between these systems have implications in the etiology and treatment of stress-related disorders and pain circuitry (142, 307, 337–340). Moreover, gonadal hormones have been shown to significantly affect visceral sensitivity in animal models, however some conflicting results have also shown no effect again highlighting the complexity of sex differences in pain processing both in a preclinical and clinical setting (173, 341–344). Interestingly, the gender of the experimenter was also shown to have a role in pain responses in preclinical models (345). Taken together, it is clear that the role of gender and/or sex in the pathophysiology of visceral pain remains a complex phenomenon which requires further investigation.

## Glutamate and Visceral Pain

### Ionotropic glutamate receptors and visceral pain

N-Methyl-D-aspartate (NMDA) receptors are not only known for their important role in excitatory synaptic transmission but also for their role in pain (346). Glutamate found in vagal and spinal afferents contributes to nociceptive signaling via NMDA and non-NMDA receptors (347). Though evidence for direct links between glutamate and visceral nociception is lacking, NMDA receptor antagonists have been shown to reduce the response of vagal and pelvic afferents in the colon and other viscera to mechanical stimuli (348). A study by Coutinho and colleagues (349) investigated the role of glutamate receptors in the RVM in visceral hyperalgesia using a colonic irritation model. They found that activation of NMDA receptors by colonic inflammation facilitated visceral hyperalgesia while non-NMDA receptors mediate inhibitory effects (349). Moreover, NMDA receptors may also be involved in the transmission of visceral nociception in the non-inflamed gut (350). It was observed that activated peripheral NMDA receptors in the colon caused the release of pro-inflammatory peptides, calcitonin gene-related peptide (CGRP) and substance-P, which have significant roles in the mediation of chronic and severe pain (350).

### Metabotropic glutamate receptors and visceral pain

Metabotropic glutamate receptors with the exception of mGlu6 receptor are expressed in all areas of the pain matrix from the spinal cord to supra-spinal sites. The action of mGlu receptors can be pro-nociceptive or anti-nociceptive depending on the subtype and site of action (351).

In the last decade, the role of mGlu receptors has gained attention in the realm of visceral pain. To our knowledge, the first study explicitly investigating the role of these receptors in visceral nociceptive processes was performed by Chen et al. (352). They found that antagonism of group 1 mGlu receptors with LY393053 reduced nociceptive behaviors in the mouse acetic acid writhing test. Furthermore, administration of a group 1 mGlu receptor agonist (S)-3,5-dihydroxyphenylglycine (DHPG) into the CeA by microdialysis increased the responses to innocuous visceral stimulation; an effect that was reversed by a reactive oxygen species (ROS) scavenger phenyl-*N-t*-butyl nitrone (PBN) and a superoxide dismutase mimetic (TEMPOL). In the same study, mGlu receptor 1 antagonist LY367385 was also found to decrease the responses to visceral stimulation (353).

Moreover, work by Lindstrom and colleagues investigated specifically the role of mGlu5 receptor in a rat model of visceral hypersensitivity (111). Here they found that mGlu5 receptor antagonism with MPEP [2-Methyl-6-(phenylethynyl)-pyridine] and MTEP [3-(2-Methyl-1,3-thiazol-4-yl)ethynyl]pyridine was sufficient to reduce the visceromotor response (VMR) in conscious Sprague-Dawley (SD) rats without altering colonic compliance. Moreover, mGlu5 receptor antagonism reduced colorectal distension (CRD)-evoked increases in heart rate and blood pressure (111). In this study, the effects seen could not be conclusively due to a peripheral or central site of action. Indeed, it has also been found that noxious colonic stimulation increases the number of Fos-positive neurons in the dorsal horn of the thoracic and lumbar spinal cord. Moreover, pre-treatment with MPEP significantly attenuated this (354).

Visceral pain originating from the bladder has also been shown to be mediated via mGlu receptors; specifically mGlu receptor 5 activation in the CeA induces bladder pain sensitization by increasing CeA output, an effect that was reversed by intra-amygdala MPEP treatment and lentivirus-mediated conditional disruption of mGlu5 receptor in the CeA (70).

### Glutamate transporters and visceral pain

Due to the negative side effects (psychomimetic activity) seen with modulation of ionotropic receptors, compounds which target these receptors are not suitable for long-term treatment of pain. To redress this and in the drive to develop better analgesics, other mechanisms such as glutamate reuptake may provide more effective treatments in controlling glutamate neurotransmission and thereby exert anti-nociceptive effects (109).

One of the first clear demonstrations that glutamate transport [via excitatory amino-acid transporters (EAATs)] may be implicated in pain processing was performed by Liaw and colleagues where they showed that selective inhibition of glutamate transporters with dl-threo-β-benzyloxyaspartate (TBOA) and dihydrokainate (DHK) produced a dose-dependent spontaneous nociceptive behavior response. These behaviors included licking, shaking, and caudally directed biting (355). Moreover, TBOA administered intrathecally was also found to induce visceral nociceptive behaviors in naïve rats (114). To further unravel the role of glutamate transport in visceral nociception, in particular stress-induced visceral hypersensitivity, the neuroprotective drug riluzole known to activate glutamate reuptake, was investigated to see whether it could attenuate visceral hypersensitivity induced by maternal separation of rats. It was found that riluzole reduces visceral hypersensitivity in stressed animals only, having no effect in non-separated animals. Moreover, EAAT-1 expression was also found to be reduced in the lumbar region of the spinal cord in hyper-sensitive animals. As mentioned previously, riluzole does not affect visceral sensitivity in naïve animals, further emphasizing the role of glutamate transport in pathological pain (114).

EAAT-2 is the main glial transporter for glutamate reuptake and its experimental over-expression in animal models has recently been found to be effective in reducing visceral pain (59, 112, 113). A study by Lin and colleagues (112) found the over-expression of EAAT-2 with the use of cephalosporin antibiotic ceftriaxone, in wildtype mice, attenuated the visceral nociceptive response to CRD. Moreover, similar effects were seen in the EAAT-2 overexpressing transgenic mouse (112). To further support these findings, systemic and intrathecal administration of DHK, a selective EAAT-2 inhibitor blocks the ceftriaxone-induced attenuation of visceral pain (112, 113). In subsequent studies performed by the same group assessing the efficacy of glutamate reuptake in animal models of colitis, it was shown that adeno-associated virus-mediated EAAT-2 over-expression was effective to mitigate VMR to 60 mmHg CRD (113). Interestingly, overexpression of EAAT-2 has been shown to reduce bladder nociception (59). However, colon irritation may affect afferents innervating the bladder thus giving a plausible mechanism for cross-organ sensitization (59). In the same study, it was also found that enhanced expression of EAAT-2 by ceftriaxone also reduced the VMR to bladder distension caused by colonic irritation (59).

As stated earlier, stress is one of the main predisposing factors for the development of visceral pain. Interestingly, glutamate transport has also been shown to be altered due to stress, with both early-life stress and stress in adulthood both showing significant alteration in EAAT expression (114, 115). Taken together, these findings provide evidence for an important role of glutamate transport in visceral pain states (271, 356). Moreover, glutamatergic signaling is critical to the process of central sensitization. This term describes the way in which excessive glutamatergic function within neurons and circuits, in particular pain circuits, leads to increases in membrane excitability and synaptic efficacy as well as reduced inhibition. These changes in synaptic function manifest as altered plasticity within the somatosensory nervous system in response to activity, inflammation, and neural injury (357).

## GABA Receptors and Visceral Pain

γ-Aminobutyric acid (GABA), the major inhibitory neurotransmitter in the CNS, plays an important role in anti-nociception. GABA is the key player acting at inhibitory synapses where it exerts its effect by binding to it respective receptors both pre- and post-synaptically. This binding causes conformational change and subsequent opening of the ion channel. The direction of the flow of ions (in/out) and their charge (+/−) result in a negative change in the transmembrane potential, causing hyperpolarization. To date, two classes of GABA receptors are known: (1) GABA_A_ receptors are ligand-gated ion channels, and (2) GABA_B_ receptors are metabotropic receptors, which are G protein-coupled receptors.

Pharmacological modulation of the GABA_A_ receptor through the use of agonists and antagonists indicate that modulation of these circuits within the spinal cord has important implications for pain processing (358–360). However, these analgesic effects particularly seen with benzodiazepines may not be purely anti-nociceptive actions and maybe more non-specific centrally mediated effects.

GABA’s anti-nociceptive effects are thought to be mediated by GABA_B_ receptors, which are ideally located in both the brain and spinal cord with ubiquitous expression. Moreover, GABA_B_ receptors have also been implicated in a whole host of GI functions, such as altering GI motility and visceral sensation (361). The mostly widely used GABA_B_ receptor agonist, baclofen has been shown to produce anti-nociception in numerous rat models of visceral pain (67, 362–365). Moreover, subcutaneous injection of baclofen was shown to prevent behavioral responses to bladder pain (362). Likewise, intrathecal administration of baclofen increases the sensitivity threshold to CRD (363). Furthermore, intravenous administration of baclofen attenuated CRD-evoked increases in arterial blood pressure and heart rate (67, 364).

Intraperitoneal injection of baclofen was shown to alter the expression of early immediate genes such as c-fos in the lumbosacral spinal cord after intra-colonic mustard oil-induced inflammation (366, 367). Moreover, it has also been shown via electrophysiological examination that GABA_B_ receptor agonists have the ability to modulate responses of vagal mucosal and muscle afferents innervating particular parts of the GI tract, specifically, the esophagus and proximal stomach (368–370). More recently, it has also been shown that baclofen dose-dependently attenuates responses of mechanosensitive pelvic nerve afferents to noxious CRD (367, 371). This data provide evidence that GABA_B_ receptor agonists may exert their positive effects by acting at peripheral sites.

However, it is important to note the effects of GABA_B_ receptor agonism, other than its anti-nociceptive effect, are undesirable centrally mediated effects, including sedation, respiratory depression, drug tolerance, and motor deficiency, thus, its potential therapeutic use could be significantly curtailed (367). Interestingly, GABA receptor pharmacology is complex with dimerization required for many subunits to form a functional receptor. Moreover, a number of splice variants of the GABA_B1_ subunit (GABA_B1a_, GABA_B1b_, GABA_B1c_, and GABA_B1d_) have been identified both in rat and human tissues, and have been found to be differentially expressed depending on the tissue type (372, 373).

Indeed GABA analogs such as gabapentin and pregabalin have also shown efficacy in preclinical models of visceral hypersensitivity (105, 106, 108, 374–377) and moreover, pregabalin has undergone clinical trials for painful CP (107). However, these compounds may exert their effects indirectly in the GABAergic system, as their main mode of action is on α2δ subunits of Ca_V_ (375). The emergence of positive allosteric modulators of GABA_B_ may provide a novel therapeutic target for treatment of visceral pain disorders (66).

## Genetic and Epigenetic Regulation of Visceral Pain

Over the last two decades, the field of pain genetics has explored the influence of genetic make-up on pain perception and processing. Recent work by Camilleri and others has described the role of genetics in IBS (378–384). Genome-wide association (GWA) studies amongst others have led to the elucidation of specific genetic alterations in IBS such as Na_v1.5_ (385), GPBAR1 (G protein-coupled bile acid receptor 1) (386), KDELR2 (KDEL endoplasmic reticulum protein retention receptor-2) and GRID2IP [glutamate receptor, ionotropic, delta 2 (Grid2) interacting protein] (381), NXPH1 (neurexophilin 1), and CDC42 (cell division control protein 42 homolog) (387). These studies are illustrative examples of how research in the genetic area can contribute to the achievement of better general knowledge in the visceral pain field.

Strain differences have been shown in many behavioral phenotypes including anxiety and depression (388, 389). Moreover, differences due to background strain have also been shown for somatic nociception (390–392). However, there is a dearth of information regarding strain differences in basal visceral sensitivity as opposed to inflammatory-induced visceral sensitivity. To our knowledge, the Wistar Kyoto (WKY) rat strain and the Flinders Sensitive Line rat strain are the only well-validated models of genetic predisposition to visceral hypersensitivity (132, 393, 394). This highlights the need for more comprehensive testing in particular assessment of visceral pain in animal models and elucidating the underlying genetic mechanisms. Moreover, the field of pain epigenetics has progressed our simplistic view of one gene, one protein, one function, to a more complex view of gene–environment interactions.

The term epigenetics refers to processes that lead to stable and/or heritable changes in gene function without any concomitant DNA sequence changes (395). Examples include DNA methylation, histone modification, and chromatin remodeling. The vast majority of work investigating epigenetic mechanisms in pain processing center around histone acetylation and DNA methylation. Pharmacological interference with the process of histone acetylation can affect pain behavior, with both systemic and intrathecal administration of histone deacetylase (HDAC) inhibitors having analgesic effects in models of inflammatory pain (396–398). In one study, this effect was shown to be mediated by expression changes of the mGlu2 receptor in both dorsal root ganglia (DRG) and spinal cord (399). Indeed, histone acetylation has been implicated in stress-induced visceral hypersensitivity and HDAC inhibitors have also shown efficacy in a model of visceral pain (69, 71, 400–402). The hypothesis that IBS could be transferred to future generations has recently been investigated (403) and discussed (404).

Similar influences could be shown in the case of DNA methylation and its reader molecule MeCP2. The methyl binding protein MeCP2 has been shown to promote abnormal up-regulation of a group of genes in inflammatory pain conditions. In rats, its usually repressive function appears to be curtailed through phosphorylation after injection of Complete Freund’s adjuvant (CFA) into the ankle joint (405), an effect thought to be partly dependent on intact descending serotonergic input into the spinal dorsal horn (398, 406). Recently, it was shown that chronic stress was associated with increased methylation of the Nr3c1 (glucocorticoid nuclear receptor) promoter and reduced expression of this gene in L6–S2 region of the spinal cord which was associated with visceral hypersensitivity in rat model (401).

Finally, recent speculation has implicated that the mechanisms and indeed pathways by which the gut microbiota may communicate with the CNS may be due in part to epigenetic processes (407).

## Experimental Models of Visceral Pain

### Colorectal distension

Colorectal distension is the most widely used method to assess visceral sensation both preclinically and clinically. This technique involves insertion of a balloon into the colorectal cavity of the human subject or animal, and with the aid of a barostat, distending in a repeated or ascending phasic manner. Although the main premise of the technique is essentially identical, the way in which it is performed varies between laboratories and researchers. This can be due to numerous factors including the model employed; whether it be human, rat, or mouse, the parameter of interest; baseline visceral sensation, pain threshold, and tolerance to painful stimuli. CRD has been characterized in humans, rats, and mice with the bulk of the work being performed in rats due to ease of use and robust reproducibility (408).

### Monitoring of visceral pain in rodents

In 1988, Ness and Gebhart were the first to describe a technique used to assess visceral sensitivity in the preclinical setting. The technique was based on the assessment of pseudoaffective and behavioral responses to controlled isobaric distensions of the GI tract. This has now become the mainstay for assessing visceral pain both clinically and preclinically (409). CRD in rats induces an array of autonomic and behavioral outputs termed pseudoaffective reflexes. These reflexes include; alterations in blood pressure and heart rate, passive avoidance behaviors, and contraction of the abdominal musculature (8), the latter of which is more commonly referred to as the VMR. VMR is the most widely used parameter of the visceral pain response (8, 408).

In the last decade, the use of electromyographic (EMG) signals and its numerous applications have allowed many to assess visceral sensitivity in conscious animals. This procedure allows recording from electrodes which are implanted in the musculature and externalized through the skin, primarily on the abdomen or neck (410–412). They can also be connected to radiotelemetric implants in the abdominal cavity (413, 414). Moreover, the development of manometric recordings measuring changes in the pressure of the balloon inserted into the GI tract, have also allowed for VMR to be assessed in freely moving animals (134, 175, 311, 408, 415, 416). Other groups have implemented other indirect approaches such as manual scoring of the abdominal withdrawal reflex (408, 417), operant behavioral assays (409), and functional brain imaging tools such as functional MRI (44, 418).

## Animal Models of Stress-Induced Visceral Pain

**Figure 5:**
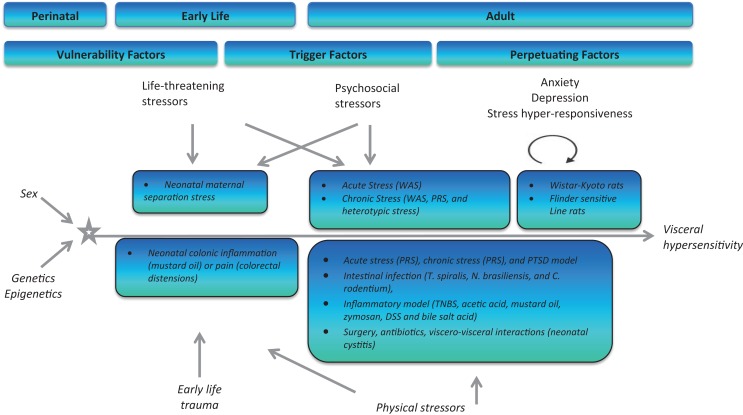
**Schematic representation of both physical and psychological stressors used in the generation of animal models for stress-induced visceral hypersensitivity**. WAS, water-avoidance stress; PRS, partial restraint stress; TNBS, trinitro benzene sulfonic acid; DSS, dextran sodium sulfate.

Stressful episodes during critical windows of development can have long-lasting effects on the host. Stress occurring during the perinatal period has been linked to the development of psychiatric disorders such as schizophrenia and autism spectrum disorders (419, 420). The early postnatal period is a stress hypo-responsive period during which time there is an intense phase of neuronal growth and myelination (421). Stress during this critical time point has been linked to the development of both somatic and psychiatric phenotypes in preclinical models, including IBS (135, 422, 423). The adolescent period is also a time of neuronal restructuring and is fundamental to precise development of the CNS (215). This developmental period is also the peak time for the onset of numerous psychiatric disorders including schizophrenia, substance abuse, and mood disorders (424). Stress in adulthood can have profound effects on its host. Both acute and chronic stressors can elicit detrimental impacts on physical as well as mental wellbeing. Taken together, it is clear that stress at critical points during our life time can have lifelong effects on the host. Overcoming the effects of these stressors is based on the individual’s HPA axis ability to adapt and overcome such insults, however, in the genetically predisposed individual, this feat maybe too high. Aberrant development of the HPA axis can prove detrimental and exhibit itself in the form of both psychiatric and somatic disorders. The importance of animal models in the search for underlying molecular mechanisms and future development of novel therapeutics has never been more pertinent (8, 425–431). Here, we review the current stress-based models in the context of IBS (Figure 5).

Experimental models of stress and stressful events have been developed bearing in mind the critical windows for HPA axis development and maturation. Animal models have been specifically developed to target different periods throughout the lifespan to assess (1) vulnerability, (2) triggering, and (3) perpetuating influences of stress and future development of IBS (425). Early-life stressors in the form of maternal neglect (maternal separation model) or injury (colonic irritation) can increase an individual’s risk to develop IBS and other disorders in adulthood (425). During adulthood, life-threatening stressors (rape, warfare), psychological stressors (acute and chronic stress), or physical stressors (in the form of intestinal dysbiosis due to infection, inflammation, antibiotic usage, and surgery) have all been described and documented as triggering factors to the development of an IBS-type phenotype in rats and mice (425). Finally, the ever expanding catalog of rodent strains and transgenic models has allowed us to use specific strains/knockouts known to exhibit various levels of stress responsivity (Wistar Kyoto and Flinders Sensitive Line) to mimic the influence of genetic and perpetuating factors on the development, severity and duration of IBS symptoms (425).

### Genetic Stress Models of Visceral Pain

Mood disorders are commonly comorbid with IBS (432–434). Genetic predisposition to such disorders has been implicated in the development of IBS in later life. These genetic factors may not lead directly to the development of IBS *per se* but may indirectly, through heightened stress responsivity, cause altered GI function and IBS symptomatology. The availability of a catalog of rodent strains has led to the advent of genetic studies assessing the exact contribution of genetic factors to disease presentation and progression. Moreover, the use of transgenic rodent strains allows us to specifically investigate single genes implicated in disease pathology.

Using different rat strains of known levels of baseline anxiety: low-anxiety SD and Fisher-344 (F344), and high-anxiety WKY rats, Gunter and colleagues were able to demonstrate a link between anxiety and visceral hypersensitivity. Specifically, high-anxiety WKY animals had increased response to CRD compared to low-anxiety strains (132). Moreover, WKY rats exhibited an exaggerated response to acetic acid instillation into the colon, a peripheral sensitization model, compared with low-anxiety strains, SD and F344 (132).

The role of the stress response and the development of IBS was again shown to be intrinsically linked when CRHR1 (CRHR1^−/−^) knockout animals were shown to have an altered VMR to CRD. VMR to CRD was only observed at the highest distension pressure (60 mmHg). Moreover, pharmacological CRHR1 antagonism decreased the VMR to CRD in CRHR1^+/+^ mice (167).

Small interfering RNA (siRNA) technologies have now become one of the most widely used approaches to selectively suppress gene expression and is a powerful tool to assess gene function. This technology has been used extensively to suppress stress-related genes in specific brain areas and models are continuously being developed (435–440). Whilst genetically modified mice have been the mainstay of behavioral genetics to date there is a growing utility for genetically modified rats (441). Moreover, other genetic tools such as optogenetic and Designer Receptor Exclusively Activated by Designer Drugs (DREADD)-based manipulations allows for both species to be used in the future (442).

### Animal Models of Early-Life Stress-Induced Visceral Pain

#### Maternal separation stress model

Stress in early life is a well-established risk factor for the development of psychiatric and somatic disorders in later life. The biopsychosocial model of IBS pathophysiology implicates adverse early-life events and childhood traumas such sexual abuse, neglect, loss of a family member, or a life-threatening situation. These factors have been linked to enhanced vulnerability of individuals to develop stress-related affective disorders such as depression and anxiety. Moreover, these individuals are also at a higher risk for developing FGIDs such as IBS and visceral pain (181, 443).

**Figure 6:**
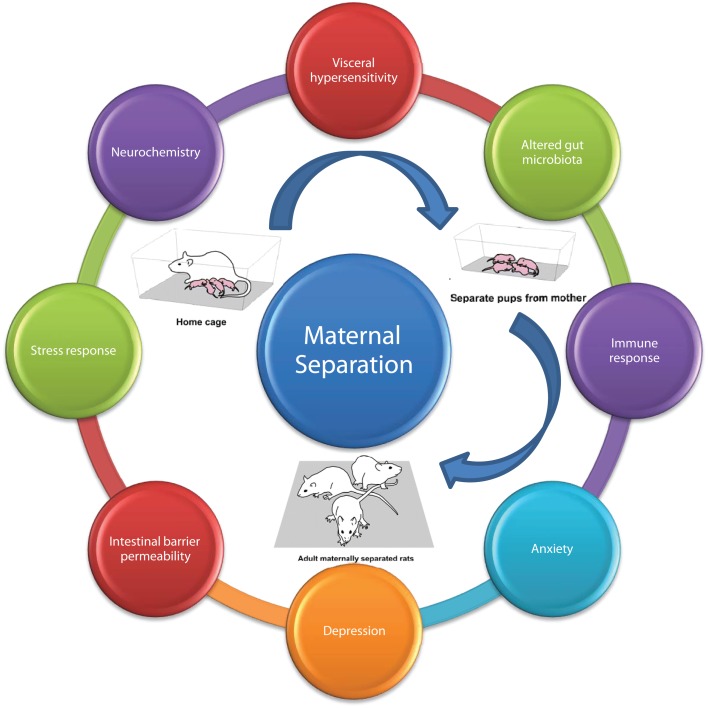
**Maternal separation model of brain–gut axis dysfunction**. Adult rodents subjected to maternal separation in early life develop the characteristic MS phenotype; typified by altered; visceral sensation, microbiota, immune response, anxiety, depression, intestinal permeability, stress response, neurochemistry adapted from Ref. (108).

To model these environmental factors in rodent models, the maternal separation model was constructed (Figure 6) (444). Briefly, this model involves removing new-born pups from the dam, during the critical HPA axis hypo-responsive phase in the early postnatal period. Most commonly, pups are removed for 2–3 h per day during the first 2 weeks of life from postnatal days 1–2 to postnatal day 12–14 (108, 188, 445, 446). This interruption in the normal maternal environment, leads to a stress response not only in the pups but also in the dams. As a result of this maternal stress response, the maternal care given to the pups is altered. In the last 20 years, the critical importance of maternal care has been researched extensively no more so in the area of epigenetics (447–449). Alterations in maternal care has been shown to have effects on the development and function of the HPA axis. Moreover, the impaired stress response is thought to underlie the deficits seen in different behavioral domains, in particular, both cognitive and emotional modalities (447). This disruption of the normal maternal environment and dam–offspring interaction can subsequently affect the quality of the maternal care received by the pups. Indeed, this model of early-life stress results in long-lasting changes to the offspring’s CNS, at all levels including altered expression, neurochemistry, electrophysiology, and morphology (8, 450).

When animals are allowed to grow to maturity, a behavioral phenotype is present, characterized by visceral hypersensitivity, increased anxiety and depressive behaviors, altered stress responsivity, altered neurochemistry specifically serotonergic neurotransmission, enhanced immune response, altered gut microbiota profile, and disruption of the intestinal barrier (108). We have previously shown that adult male rats previously subjected to maternal separation exhibit visceral hypersensitivity to CRD (135). Moreover, the effects of MS are exacerbated by exposure to an acute stressor specifically 1 h of water-avoidance stress (WAS) (451, 452).

The maternal separation model is sensitive to many factors such as sex differences, with the abundance of studies being performed in male rodents, however clinically the preponderance of female to male IBS patients is 3:1. It is unclear from the literature that the MS procedure induces the same robust phenotype in female rodents. Moreover, the protocol of separation itself has also proved crucial. Specifically, sex-dependent effects on VMR were evident when the separation was performed by the removal of an entire or half litter from the home cage (446). Males that were exposed to whole litter separation and females that were exposed to both whole litter and half litter separation developed visceral allodynia and hypersensitivity to CRD (446). Moreover, when males underwent an additional acute stress, this did not modify the CRD response. On the other hand, when females were exposed to an additional acute stress, they exhibited an exacerbated response to CRD (8, 446). The rat MS model is the most commonly used rodent model of IBS and has proved crucial in delineating the underlying mechanisms as well as testing novel therapeutic strategies.

Although the maternal separation model is well established and characterized in rats, its utility in mouse strains has proved difficult to replicate. Data from our lab and others suggest that maternal separation stress alone is insufficient to induce a robust, reproducible behavioral phenotype (453, 454). However, when maternal separation stress is combined with unpredictable maternal stress, a more overt behavioral phenotype is exhibited (310, 455).

#### Neonatal Irritation Models

Early-life stress not only in the form of psychological stress but also in the context of physical stress has been shown to be a valid preclinical model of IBS pathophysiology. The neonatal intestine when exposed to mechanical and chemical stressors results in a pro-inflammatory phenotype with mucosal inflammation and tissue irritation. Daily irritation of the neonatal colon by mechanical irritation (daily noxious CRD) or chemical irritation (daily intra-colonic injection of mustard oil) increases pain behaviors in response to CRD from adolescence to adulthood (417, 456). Moreover, this heightened pain response was accompanied with decreases in exploratory behavior, indicative of an anxiety-phenotype. The findings described in these studies implies that irritation of the neonatal gut, be it via mechanical or chemical means, can lead to the process of central sensitization due in part to sensitized peripheral afferents (8, 417, 456). These behavioral outputs are in parallel with IBS in the clinical scenario; visceral hypersensitivity with comorbid anxiety.

### Animal Models of Adult Stress-Induced Visceral Pain

#### Acute stress models

Acute stressors in adulthood can lead to an immediate behavioral phenotype. The most widely used acute stress paradigms to model IBS preclinically are WAS and restraint stress. The WAS paradigm was originally developed by Bonaz and Tache and Enck et al. (457, 458) to assess stress-related alterations in gut motility and motor function. Briefly, rodents are placed on a small platform raised slightly above water level. This stressor is based on the aversive environment of surrounding water. The WAS paradigm has been used extensively to assess the impact of psychological stress on visceral pain modulation (8, 311). Data from the literature show that 1 h WAS was sufficient to induce a delayed visceral hypersensitivity to CRD, in male Wistar rats (459). Moreover, others have shown that chronic WAS-induced effects are thought to be modulated at an epigenetic level, with HDAC inhibitors shown to reverse WAS-induced hyperalgesia (400).

Moreover, other forms of acute stress such as restraint stress for 2 h, was also found to induce heightened VMR to CRD in male (460) and female Wistar rats (446). The time spent in an acute stress situation has proven crucial to the development of the IBS phenotype (461). Acute stressors of longer duration >2 h tend to be susceptible to the animals habituating to the stressful environment and this can confound the behavioral outputs. Moreover, WAS was recently shown to induce both hyperalgesia and analgesia depending on the duration and number of stress sessions (311, 462). Transient stressors generally trigger adaptive responses and this type of model mimics the stress-related hypersensitivity to CRD as reported in IBS patients.

#### Chronic mild stress

Daily life stressors affect individuals in different ways. Persistent mild stressors can accumulate and potentiate the effects of stress on the host. Modeling chronic daily stress in rodents is achieved through the chronic mild stress (CMS) paradigm. Convergent lines of evidence suggests that high levels of chronic daily stress can impact on the intensity and severity of visceral pain symptoms (181, 463–466). In light of this, an array of rodent models involving unpredictable chronic and intermittent exposure to variety of stressors have recently been developed (8). Following on from the previous section, WAS was in fact one of the first chronic stress models to be adapted to the study of visceral hypersensitivity (467). Early data proved promising with studies showing that a 1 h daily WAS, for 10 consecutive day protocol was sufficient to induce visceral hypersensitivity to CRD in male Wistar rats (410, 468). This effect was long-lasting and persistent up to 30 days after the end of the stress. One of the confounds of this study was based on the methodology used to assess VMR. EMG recordings were performed however this involves surgical implantation of electrodes and ensuing single housing of animals, to avoid injury (410), which in itself could be described as a stressor. Indeed, when VMR was assessed using intraluminal colonic solid-state manometry, both male and female naïve Wistar rats exposed to WAS developed a reduced response to CRD referred to as visceral analgesia (311). This findings appear to be in direct contrast to each other and highlight the challenges of developing models of stress-induced visceral hypersensitivity.

Moreover, the picture becomes even more complex when we consider mouse models. Indeed, chronic WAS in C57Bl/6 mice has shown many varied effects including visceral hyperalgesia (311), visceral analgesia (311), or to have no influence on the VMR (469). Many other factors may be of course at play such as the conditions prior to CRD including surgery and housing environment, and these factors are dependent on the route of VMR assessment (311). Taken together, it becomes clear that the basal environment and state of the animals prior to stress exposure can impact their response to the stressor itself (8).

Furthermore, the nature or type of stressor is also crucial, with habituation known to occur when animals are repeatedly exposed to the same type of stressor, a phenomenon thought to be mediated via oxytocin (470) and endocannabinoid pathways (471). Moreover, these pathways are indeed themselves also important in pain processing (472, 473). Taking this knowledge on board, that homotypic stressors may/do lead to habituation, more recent models employ stressors of different natures. These heterotypic stressors such as cold restraint stress, WAS, or forced swimming were found to induce immediate but not long-term visceral hyperalgesia as monitored by EMG recordings at 8 h, 24 h, and 7 days after the cessation of the stress paradigm in male Wistar rats (474). Developing the model further, with more multifaceted paradigms and longer lasting stress sessions (134, 475, 476) causes a behavioral phenotype in rodents that mimics aspects of depression. This more recently developed model may prove useful when assessing the mechanisms of chronic visceral pain comorbid with depression-like symptoms (432). Taken together, it is clear that many other factors outside of the stress model can have significant effects on the parameter of interest and thus limit the models utility (477). Factors such as the sex and strain of the animal model, housing conditions before, during and after the stress paradigm and diurnal variation are all known to alter sensitivity to stress (477). From the literature, what we have learned is that variety within the stress paradigm itself (time, type of stressor) appears crucial to inducing stress-induced effects and preventing habituation (470, 478–480). All of these facets could also in themselves potentially alter the influence of CMS paradigms on the visceral pain sensitivity, as recently assessed by Larauche et al. (311).

#### Chronic psychosocial stress

Daily stressful events are commonplace in modern society. However, when modeling stress in rodent models, the stressors most often occur in a novel environment and not in the animals’ home cage. This has been a cause for critique with some of the most widely used stress models. The chronic social defeat and overcrowding paradigm was designed to overcome this obstacle and thus animals undergo stress scenarios in their home cage. The paradigm is based on the unpredictable nature of life’s stressors. Sessions of resident intruder social defeat and cage overcrowding are randomized so as to occur at different times of the day, in an unpredictable manner and for a chronic period, 19 days in total. We have recently shown this model to be an effective preclinical model to mimic many of the key phenotypes in the IBS population, specifically a heightened response to CRD (134) and anxiety- and depression-related behaviors (481).

Moreover, Reber and colleagues (482) have shown that this model of chronic psychosocial stress robustly enhances GI dysfunction in a mouse model of colitis. Furthermore, other chronic psychosocial stressors such as chronic overcrowding when applied to rats also induces a heightened sensitivity to CRD concomitant with enhanced HPA axis activity and intestinal mucosal inflammation (483). Taken together, these studies demonstrate that this model of chronic psychosocial stress may have multiple effects across the brain–gut axis resulting in an IBS phenotype.

#### Conditioned fear-induced stress model

There is increasing evidence of augmented prevalence of GI symptoms, including visceral pain and IBS in patients suffering from post-traumatic stress disorder (PTSD) (122, 484–487). Indeed, it has been shown that in some individuals, the experience of stress can prime such individuals to respond differently to subsequent stressful events. Over the last decade, Stam and colleagues have investigated this in preclinical models, particularly rats. They have shown that short exposures to shocks or a social confrontation environment with a predator or aggressive conspecific animal induces long-lasting conditioned fear-responses to trauma-related cues (425). Moreover, these animals exhibit a generalized behavioral sensitization to novel stressful stimuli that is persistent and may intensify over time (488–491). Furthermore, this group have also shown that 2 weeks after a single session of foot shocks, repeated CRD causes increased cardiovascular reflexes in pre-shocked rats when compared to their non-shocked controls (489). Remarkably, female rats appear to exhibit an alternative pattern of sensitized behavioral responsiveness to the same challenge, again highlighted the strong role of sex differences in visceral pain processing (492). This specific rodent model mimics clinical features of a subset of IBS patients exhibiting stress-related visceral hypersensitivity due to PTSD. However, it is important to note that the findings presented above are mainly characterized by a single group of investigators, thus the reliability of the model needs further independent confirmation.

#### Physical stressors

##### Post-infectious models of visceral pain

A significant proportion of IBS cases occur after an illness particularly an infection of the GI tract. This is reported to be as much as 10% of patients with IBS (493). Interestingly, the proportion of people who suffer from an intestinal infection who will then go on to develop IBS ranges from 3 to 36% and appears to be dependent upon the infecting organism. Moreover, the psychological state of the individual at the time of infection appears to also play a critical role in the development of IBS symptoms (494). Infections of bacterial origin when compared to the short-lived effects of viral gastroenteritis appear to be more long-lasting and persistent (494). A transient *Trichinella spiralis* infection was shown to induce sustained visceral hypersensitivity in a mouse model (5, 495). Moreover, similar findings were found in a rat model of *Nippostrongylus brasiliensis* infection (496). Although the vast majority of human post-infectious hypersensitivity symptoms are observed after bacterial infection with *Salmonella, Escherichia coli*, *Shigella*, or *Campylobacter*, there has been limited animal models of this type of visceral hypersensitivity (497, 498).

##### Post-inflammatory models of visceral pain

Inflammation is one of the leading causes/mechanisms thought to underpin IBS and its associated symptomatology (499–502). However, this remains a controversial topic and others have reviewed this previously (503, 504). Moreover, this symptom set appears to be common in patients of inflammatory bowel disorders also such as those in remission from ulcerative colitis (505). Indeed, many clinical cases of IBD switch to IBS cases and this occurs in 30–50% of patients (506). Inflammatory pain models have been well developed in somatic pain assays and to some extinct in visceral pain models. Indeed, an array of chemical irritants have been used in preclinical models to induce colonic inflammation and resultant visceral hypersensitivity (8). In rats, mustard oil (507, 508), acetic acid (509), and zymosan (510, 511) have been shown to induce short-term visceral hypersensitivity due in part to colonic inflammation. Moreover, other compounds such as TNBS induce severe colonic inflammation and visceral hypersensitivity when administered intra-colonically (512, 513). Interestingly, in some models of inflammatory-induced visceral hypersensitivity, the phenotype can re-emerge days or weeks after the initial inflammatory response. Moreover this re-emergence is not associated with any inflammatory biomarkers (512, 514). Importantly, the experimental design appears to play a major role in the application and effectiveness of such models. Models, such as the dextran sodium sulfate (DSS)-induced colitis model has provided us with some surprising findings where animals exhibited an increased response to CRD on day 5 or day 60 after the induction of colitis in male Balb/c mice. However, chronic DSS colitis was not associated with changes in VMR to CRD (515). However, contrasting findings have also been reported by other groups, whereby DSS colitis failed to induce any alterations in VMR or visceral sensitivity at all time points tested in either C57Bl/6 or Balb/c mice (516). One can speculate on the many possible reasons for these observed differences, however, what they do suggest is that inflammation on its own may not be sufficient to induce visceral hypersensitivity and that the nature and severity of the inflammatory stimulus and their combined effects may determine when/whether post-inflammatory hypersensitivity will result if at all (8, 512). Moreover, as is seen in post-infectious visceral hypersensitivity, psychological state may also play a role (517). Indeed this was investigated by Larsson et al. (469), who demonstrated that prior exposure of animals to a stressor, be it either psychological or psychosocial in origin, revealed an enhanced susceptibility to colitis and an aggravated colonic inflammatory response (482, 518, 519). Furthermore, this was also associated with a heightened susceptibility to recurrence of colonic inflammation even though the colitis had dissipated (520, 521). Similarly, a prior bout of colitis was sufficient to leave the colon in a state more susceptible to the negative effects of stress (522). However, the complex association between colitis, stress, and visceral pain response remains contentious as stress has also been found to both exacerbate or indeed to have no effect on post-inflammatory visceral sensitivity in rats (523) and in mice (8, 469).

## Future Directions

### Microbiota manipulations

The role of the microbiota–brain–gut axis on health and disease is an area of biomedicine that is receiving much media attention of late. The complex communication between the gut microbial population and the CNS has far reaching implications not least in the area of visceral pain and psychiatric comorbidities. Modulation of the gut microbiota via prebiotic/probiotic treatment has been shown to have positive effects on visceral pain behaviors as discussed earlier (222, 240–242). Antibiotic treatment has long been known to alter GI function, effects which are reversed upon probiotic treatment (524). Thus, it is logical that antibiotic-induced visceral pain may be a future model used to investigate the underlying mechanism of such a phenomenon. Indeed work from our own lab has recently demonstrated that early-life antibiotic-induced disruption of the colonizing microbiota is associated with visceral hypersensitivity and altered spinal signaling in adulthood. These results indicate that a temporary alteration in the composition of the GI microbiota during a crucial time-window in the neonatal rat has a long-lasting impact on nociceptive pathways and that the developing pain systems are subject to modulation by the microbiota (248).

Moreover, intriguing studies to assess the exact contribution of the gut microbiota to the development of visceral hypersensitivity and IBS can be achieved through fecal transplantation studies whereby fecal matter from donor IBS patients can be used to inoculate recipient animals and subsequent behaviors can be assessed. This paradigm has already shown its merit in transferring other behaviors specifically anxiety-related behaviors in a mouse model (525).

## Conclusion

An effective stress response is critical to the survival of any living creature. The ability to sense changes in the environment and adapt accordingly is quintessence to survival. However, the host’s reaction to perceived stress can sometimes become disturbed leading to an over-exaggerated response. Stress has been implicated in and associated with a plethora of somatic disorders; ulcers, migraine, hypertension, and psychiatric disorders; anxiety, depression, PTSD. Here, we specifically reviewed the literature in the context of stress as a major risk factor for the development of IBS and visceral pain. The mechanism by which stress impacts on normal physiology to cause such devastating disorders still remains unclear. Numerous mechanisms have been postulated including alterations in plasticity, neurogenesis, catecholaminergic neurotransmission, and more recently gut microbiota. Modern day life exposes us all to stressors of varying types (psychological/physical) and severities (acute/chronic) and as such the search for novel pharmaco-therapeutics has never been more pertinent.

Despite the ever growing body of literature, the exact mechanisms underlying visceral pain still remain less well understood than that of somatic pain. Central sensitization of primary sensory afferents is an important underlying mechanism for both somatic and visceral hypersensitivity and hyperalgesia. One of the hurdles in understanding the mechanisms of stress-induced visceral pain is that visceral pain response is experienced differentially depending on numerous factors, including the time at which stress was applied, duration of stress, sex differences, and genetic background amongst others. Psychological stress at any point during our lifetime can lead to permanent alterations of the HPA axis, the descending pain modulatory system, the immune system, and the gut microbiota, all of which can be manifested as chronic visceral hypersensitivity. Mechanisms by which physical stress such as infections, mediate visceral hypersensitivity are likely to be different to that of psychological stress and may involve altered immune system functioning. Considering the diverse mechanisms of visceral pain, the development of treatment strategies and therapeutic interventions will rely on good animal models, all of which have been reviewed here. It is clear that that the drive to develop clinically relevant models and thus design new novel therapeutics has never been more pertinent.

## Conflict of Interest Statement

The authors declare that the research was conducted in the absence of any commercial or financial relationships that could be construed as a potential conflict of interest.
